# New concepts on the etiology of endometriosis

**DOI:** 10.1111/jog.15549

**Published:** 2023-02-06

**Authors:** Fiona L. Cousins, Brett D. McKinnon, Sally Mortlock, Harriet C. Fitzgerald, Chenyu Zhang, Grant W. Montgomery, Caroline E. Gargett

**Affiliations:** ^1^ The Ritchie Centre Hudson Institute of Medical Research Clayton Victoria Australia; ^2^ Department of Obstetrics and Gynaecology Monash University Clayton Victoria Australia; ^3^ Institute for Molecular Biosciences, The University of Queensland Brisbane Australia

**Keywords:** endometrial stem/progenitor cells, endometriosis, genetic variants, retrograde menstruation, somatic mutations

## Abstract

Endometriosis is a serious, chronic disorder where endometrial tissue grows outside the uterus, causing severe pelvic pain and infertility. It affects 11% of women. Endometriosis is a multifactorial disorder of unclear etiology, although retrograde menstruation plays a major role. It has a genetic component with over 40 genetic risk factors mapped, although their mechanism of action is still emerging. New evidence suggests a role for retrograde menstruation of endometrial stem/progenitor cells, now that identifying markers of these cells are available. Recent lineage tracing and tissue clearing microscopy and 3D reconstruction has provided new understanding of endometrial glandular structure, particularly the horizontal orientation and interconnection of basalis glands. New sequencing technologies, particularly whole genome DNA sequencing are revealing somatic mutations, including in cancer driver genes, in normal and eutopic endometrium of patients with endometriosis, as well as ectopic endometriotic lesions. Methylome sequencing is offering insight into the regulation of genes and the role of the environmental factors. Single cell RNA sequencing reveals the transcriptome of individual endometrial cells, shedding new light on the diversity and range of cellular subpopulations of the major cell types present in the endometrium and in endometriotic lesions. New endometrial epithelial organoid cultures replicating glandular epithelium are providing tractable models for studying endometriosis. Organoids derived from menstrual fluid offer a non‐invasive source of endometrial tissue and a new avenue for testing drugs and developing personalized medicine for treating endometriosis. These new approaches are rapidly advancing our understanding of endometriosis etiology.

## INTRODUCTION

### Endometriosis and disease heterogeneity

Endometriosis is defined as the presence of endometrial‐like tissue outside of the uterine cavity. Endometriotic lesions can form on the peritoneal surface of body organs, including the bowel, ovaries, uterus, bladder, on the body wall lining the peritoneal cavity and deep infiltrating lesions form in the pouch of Douglas. Endometriosis lesions cause a multitude of symptoms; chronic pelvic pain, bowel and bladder dysfunction, painful sex. Approximately 30%–50%^1^, ^2^ of patients will be diagnosed with infertility and approximately 50% will suffer from anxiety or depression.^3^

Lesions form close to blood vessels, ensuring their survival, and become highly innervated contributing to the chronic pelvic pain experienced by many sufferers. Persistent inflammation in the peritoneal cavity resulting from repetitive deposition of menstrual tissue and active breakdown of established lesions, also contributes to pain and allows lesions to persist in the peritoneal cavity.

Endometriosis research, diagnosis, treatment and management is complicated by the heterogeneity of the disease.^4^ The number or location of lesions does not correlate with symptoms^5^ and for some patients, the disease is discovered when they undergo explorative laparoscopy for unexplained infertility. Lesions appear in different forms as they progress from new clear/white, to red, then black lesions^6^, ^7^ and finally a white scar reflecting a loss of endometrial glands and stroma and increased collagen deposition.^8^ However, macroscopically similar lesions have different behaviors and can cause different symptoms.^4^ Lesion morphology is also heterogenous. In a study of superficial lesions, inter‐ and intra‐patient variability of gland profiles and stroma was independent of menstrual cycle stage^9^ with the authors suggesting that the different gland profiles may reflect the lesions' responses to steroid hormones. This is supported by another study where deep infiltrating lesions exhibited high variability of estrogen receptor alpha and progesterone within glands, and between lesions and patients.^10^ This may explain why patients have variable responses to hormonal therapies, highlighting the need to personalize therapy for each patient.

### Theories of endometriosis etiology

#### Retrograde menstruation

**FIGURE 1:**
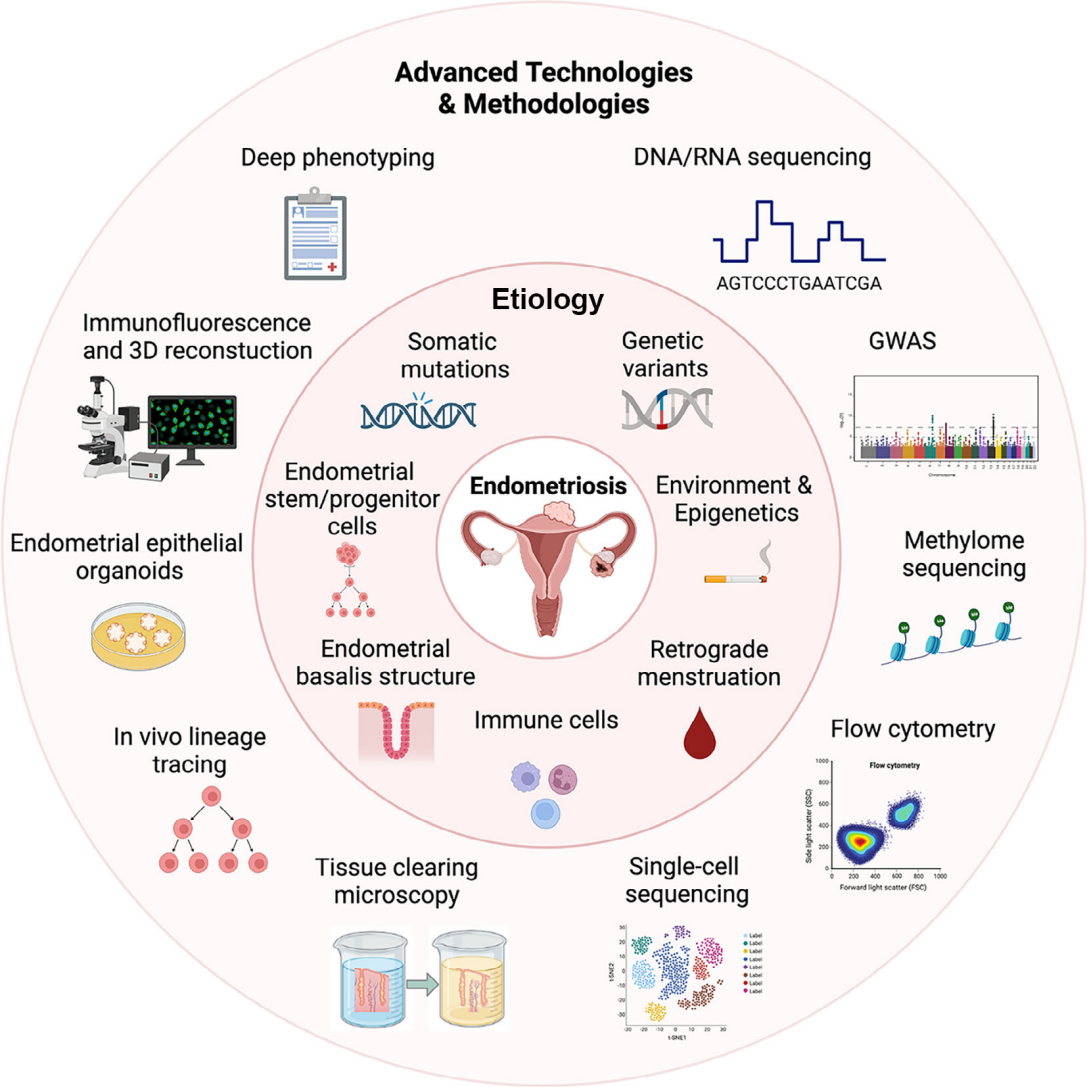
Schematic showing that advanced technologies and methodologies are enabling the generation of new concepts on the etiology of endometriosis.

Sampson^11^ was the first to hypothesize that retrograde menstruation may cause endometriosis (Figure 1). According to his theory, menstrual blood refluxes backwards through the Fallopian tubes into the pelvic cavity, whereby menstrual tissue fragments attach to peritoneal organs and develop lesions. Retrograde menstruation is found in over 90% of menstruating patients during gynecological surgery^12^ and endometrial stem/progenitor cells have been isolated from peritoneal fluid^13^ suggesting a potential mechanism for their survival and differentiation in endometriosis patients^14^ (see below). Young people with oblique vaginal septum syndrome, cervical atresia, and other obstructive genital tract malformations are more likely to have endometriosis,^15^, ^16^ supporting a role for refluxed menstrual fluid reaching the peritoneal cavity. The asymmetrical anatomical distribution of superficial endometriosis lesions, with a greater proportion located on the right‐hand side of the peritoneal cavity due to clockwise peritoneal currents, also supports retrograde menstruation.^17^, ^18^ While 90% of menstruators may experience retrograde menstruation, the overall prevalence of endometriosis approximates 11%,^19^ indicating additional factors are involved in endometriosis pathogenesis.

#### Additional etiologies of endometriosis

Sampson's theory fails to explain the etiology of endometriosis in males,^20^ and females with Mayer–Rokitansky–Küster–Hauser syndrome.^21^, ^22^, ^23^ The Mullerian remnants hypothesis implies that endometrial‐like tissue in the peritoneal cavity are derived from primitive endometrial cells misplaced during embryonic development.^24^ The coelomic metaplasia hypothesis suggests that the coelomic epithelium, which gives rise to Mullerian duct epithelium, the precursor cells of the endometrium, is the source of cells seeded during development. They lie dormant until exposure to endogenous hormones at menarche.^25^ Multiple origins may contribute to endometriosis, especially given the disease heterogeneity. However, evidence from clinical and molecular studies, especially recent observations on patterns of somatic mutations in endometrium and lesions^26^, ^27^, ^28^ provides strong support for retrograde menstruation as the most common source of cells for endometriosis lesions.^29^, ^30^

Endometriosis is multifactorial in etiology, with genetics and environment each contributing approximately 50%.^30^ The cells of origin of endometriosis lesions are likely stem/progenitor cells as only these cells are clonogenic and have the ability to initiate new growths of endometrial tissue in ectopic sites.^31^ Clonogenic endometrial cells are found in specific niches, mainly, but not exclusively, in the basalis endometrium.

## NEW CONCEPTS ON THE ETIOLOGY OF ENDOMETRIOSIS

### Structural features of the endometrial basalis

The endometrium undergoes over 400 menstrual cycles during a woman's reproductive lifespan. Basic histological techniques first described post‐menstrual endometrial epithelium migrating from the stumps of the basalis glands of menstruating endometrium.^32^, ^33^ These findings were confirmed by scanning electron microscopy^34^ and hysteroscopy.^35^ Markee highlighted vascular changes and the speed of luminal epithelial repair by transplanting the endometrium into the eye of rhesus monkeys.^36^ More recently the structure of the basalis layer was revealed by lineage tracing of mitochondrial DNA mutations and 3D reconstruction of fixed tissue (Figure 1). This study identified complex interconnected horizontal basalis glands as the origin of non‐branching, single, vertical functionalis glands.^37^ The authors also found evidence of a multipotent epithelial stem/progenitor cell that regenerated the basalis and functionalis glandular lineages. Others have identified somatic mutations in basalis epithelial cells that suggest endometrial glands arise from a single ancestral cell.^28^ Tissue clearing microscopy, immunofluorescence and 3D reconstruction confirmed the horizontal rhizome‐like glandular network (Figure 1)^38^ and showed the spatiotemporal dynamics of glands in human endometrium.^39^ They also showed that multiple vertical glands originate from a single ancestral clone in a horizontal segment of a basalis gland, and that the vertical glands diversify by acquiring additional mutations. This horizontal rhizome‐like structure of endometrial basalis glands may safeguard them from enzymatic destruction during menstruation thereby protecting the epithelial progenitor cell niche.

### Endometrial stem/progenitor cells

**FIGURE 2:**
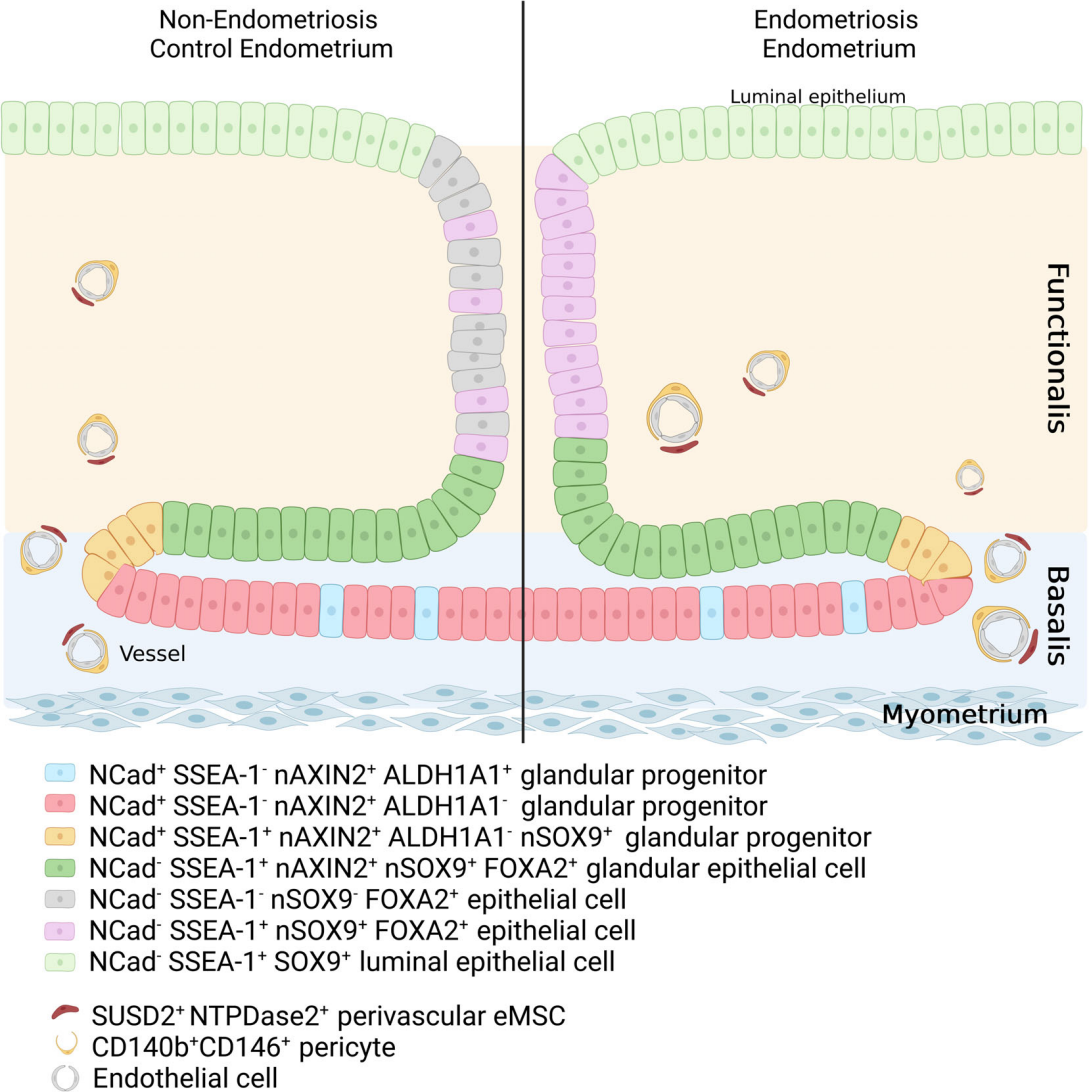
Schematic showing the location of endometrial stem/progenitor cells in the basalis, functionalis and luminal epithelium of human endometrium based on specific surface markers of these cells demonstrating adult stem cell activity. Note the horizontal gland structure in the basalis gland from which emanates the vertical gland in the functionalis. It is likely that the NCAD^−^SSEA‐1^+^nSOX9^+^basalis epithelial cells re‐epithelialize the raw endometrial surface during menstruation to become the luminal epithelial cells. In endometriosis, most functionalis glandular epithelial cells are SSEA‐1^+^nSOX9^+^ (purple, right hand side), in contrast to normal endometrium, where they are only occasionally found in the functionalis (purple, left hand side). CD140b, PDGFRβ; eMSC, endometrial mesenchymal stem cells; NCAD, N‐cadherin; NTPDase2, nucleoside triphosphate diphosphohydrolase‐2 *Source*: Adapted from Salamonsen et al.^42^ and Cousins et al.^43^

Human endometrial stem/progenitor cells were first identified as clonogenic cells,^40^ which demonstrated adult stem cell properties of self‐renewal, differentiation and high proliferative capacity in in vitro functional assays.^41^ Both epithelial progenitor cells and mesenchymal stem cells (eMSC) were identified. Surface markers were discovered for both stem/progenitor cell types that enriched for the respective clonogenic cells and also demonstrated the above classic stem cell properties (Figure 2). Specifically, co‐expression of PDGFRβ and CD146 isolated a small population of eMSC, demonstrating their pericyte identity by immunofluorescence.^44^ A single perivascular marker, SUSD2 (formerly W5C5), also purifies clonogenic eMSC,^45^ which reconstitute stromal tissue in vivo. Unbiased gene expression profiling of highly purified endometrial epithelial cells, comparing pre‐menopausal and post‐menopausal endometrium, found 11 differentially upregulated surface markers in post‐menopausal cells.^46^ The most consistent was *CDH2*, which encodes for N‐cadherin (Figure 2). Magnetic bead‐sorted N‐cadherin^+^ endometrial epithelial cells were enriched in clonogenic cells compared to N‐cadherin^−^ cells, and demonstrated adult stem cell properties in functional in vitro assays. N‐cadherin^+^ epithelial cells were located in the deepest gland profiles in the rhizome‐like glands of the basalis, directly adjacent to the myometrium. The surface marker, SSEA‐1 identifies basalis epithelium (Figure 2) and has the progenitor properties of longer telomeres, telomerase activity and differentiation in in vitro assays.^47^ These markers highlighted a potential cellular hierarchy in human endometrium (Figure 2) with the most primitive N‐cadherin^+^ progenitors located in the bases of the horizontal glands and the SSEA‐1^+^ cells proximal to the N‐cadherin^+^ cells.^46^ SSEA‐1^+^ cells are located at the ill‐defined basalis‐functionalis junction indicating they would migrate from the gland stumps during menstruation to rapidly resurface the denuded endometrial surface to become the new luminal epithelium.^42^ Two transcription factors, nuclear AXIN2^48^ and nuclear SOX9^47^ also selectively mark basalis epithelium (Figure 2), although recent spatial transcriptomics also found *SOX9* in the functionalis of proliferative stage endometrium.^49^ These markers of human endometrial stem/progenitor cells have enabled their quantification in endometrial tissues and body fluids.^13^

Sampson's theory of endometriosis has now been extended to include endometrial stem/progenitor cells (Figure 1) which have been found in menstrual fluid as N‐Cadherin^+^ cells and SUSD2^+^ eMSC.^13^ Importantly, these cells were found in peritoneal fluid on day 2–3 of menstruation in people with endometriosis, but also in a small proportion of people without the disease. Endometrial stem/progenitor cells were occasionally found in peritoneal fluid of non‐menstruating people with endometriosis, but not in controls, suggesting persistence of stem/progenitor cells with potential to initiate lesions. Clonogenic cells were also found in menstrual and peritoneal fluids of menstruating people, but not in peripheral blood, indicating their source is endometrium rather than bone marrow.^13^ SSEA‐1^+^ epithelial cells were found abnormally in the glands of functionalis endometrium of people with endometriosis,^50^ suggesting they will be shed in large numbers in menstrual fluid where they may gain access to peritoneal cavity during menstruation. More recently, SSEA‐1^+^ cells were found in menstrual fluid.^51^ This raises the question of whether luminal epithelial SSEA‐1^+^SOX9^+^ cells retain progenitor cell activity as they also reach the peritoneal cavity during menstruation. Together, these findings suggest that genetic and environmental endometriosis risk factors may differentially affect retrogradely shed endometrial epithelial progenitor cells, the likely cells of origin of endometriosis lesions, to promote the establishment of lesions in women with endometriosis. Clonogenic stomal cells have been identified in endometriosis lesions.^52^ Another source of endometrial stem/progenitor cells in peritoneal fluid during menstruation could be any shed from established superficial endometriotic lesions. Thus, susceptibility to forming lesions in women with endometriosis may be the presence of endometriosis risk genes, somatic mutations and/or environmentally‐mediated epigenetic changes in the stem/progenitor cells that provide a competitive advantage for lesion initiation.

Upon reaching the pelvic cavity, clonogenic endometrial stem/progenitor cells need to adhere to the mesothelial lining and/or invade beneath the peritoneum to establish an endometriotic lesion.^53^ Since N‐cadherin^+^ epithelial progenitors express nuclear ERα,^46^ it is expected they would respond to rising estrogen levels during the proliferative stage of subsequent menstrual cycles to generate the ectopic glands in developing endometriotic lesions. Some SSEA‐1^+^ basalis epithelial cells show nuclear ERα in 3D cultures,^47^ suggesting they may also directly respond to estrogen to proliferate and generate lesions. In contrast, the ERα^−^ SUSD2^+^ eMSC^54^ rely on ERα‐expressing niche cells (endothelial, perivascular or stromal) in eutopic and ectopic endometrium to respond to estrogen. eMSC signal in a paracrine manner to endothelial cells by releasing of angiogenic growth factors^55^ and promoting angiogenesis required for lesion growth.

In stromal endometriosis, SUSD2^+^ eMSC in menstrual fragments containing niche cells may attach and initiate stromal endometriosis lesions when shed into the pelvic cavity. Here they may proliferate and differentiate into endometrial stromal cells to form endometriotic lesions, as we have shown previously in mouse transplantation studies.^45^ This is supported by gene profiling studies demonstrating that CD146^+^PDGFRβ^+^ eMSC from women with endometriosis spontaneously differentiate into stromal cells in vitro and pass on a decidualization defect to their cellular progeny, not present in normal eMSC or stromal cells.^56^, ^57^ Single cell RNA sequencing (scRNAseq) of fresh eutopic endometrium, peritoneal lesions and adjacent peritoneal tissue showed many *SUSD2* ^+^ cells with a perivascular, angiogenic and immunomodulatory gene expression profile indicating their key role in peritoneal lesion progression.^58^ However, scRNAseq studies on concurrent fresh menstrual and peritoneal fluid samples are needed to confirm that eMSC are the cell of origin to establish endometriotic stromal lesions in vivo.

### Retrograde endometrial waves to transport cells of origin of endometriosis into the pelvic cavity

The Müllerian Duct‐derived inner myometrium, comprising circular smooth muscle cells, undergoes abnormal caudal‐fundal‐directed contractions producing endometrial waves that transport endometrial tissue fragments into the pelvic cavity of menstruating women with endometriosis with greater frequency than control women.^59^, ^60^, ^61^ This uterine dysperistalsis implicated in abnormal retrograde menstruation, has previously been assessed by transvaginal ultrasound videos or transport of radioactive particles to determine direction of uterine contractions,^62^, ^63^ however these approaches are subjective and laborious. Recently introduced electrohysterography used to monitor pregnancy,^64^ provides a new more quantitative and less invasive technique that could be adapted for determining the role of abnormal endometrial waves associated with retrograde menstruation in women with endometriosis.^65^

### Endometrial‐mesenchymal‐transition and mesenchymal‐epithelial‐transition

Epithelial‐mesenchymal‐transition (EMT), the process where epithelial cells slowly lose their epithelial phenotype and gain a mesenchymal phenotype, has been implicated in progression but not establishment of endometriosis. Several studies comparing eutopic endometrium from control and endometriosis patients revealed only subtle differences in EMT specific‐pathway markers, suggesting that EMT may not be involved in disease initiation,^66^ reviewed by Konrad et al.

Increased protein expression of EMT pathway markers TWIST,^67^ SNAIL,^68^, ^69^ SLUG^70^ and ZEB1^71^ and mesenchymal marker N‐cadherin^72^ were observed concurrently with decrease expression of the epithelial marker, E‐cadherin, in ectopic lesions compared to patient matched eutopic endometrium.^70^ Interestingly, E cadherin expression was higher in deep infiltrating lesions compared to ovarian and peritoneal lesions,^72^ in keeping with the hypothesis that mesenchymal‐epithelial‐transition (MET) occurs in deep infiltrating endometriosis.^72^ MET has also been implicated in the progression of red lesions to black lesions via epithelial cell differentiation.^73^, ^74^ Cell–cell contact markers, Claudins 1 and 4 have decreased protein expression in lesions^75^ but other Claudins are unchanged, suggesting that cell–cell contacts remain intact in ectopic lesions and that only partial EMT occurs. While location of lesions was assessed in many studies, the stage of disease was not, therefore it is difficult to ascertain whether partial EMT may be completed as disease progresses or whether the epithelial cells maintain a partial EMT state throughout the life of a lesion. Approximately 50% of endometriotic lesions contain glandular epithelium when examined histologically^76^ but, due to limitations with longitudinal studies of lesions, it is unknown whether a menstrual fragment needs an epithelial compartment to survive and grow, or whether the glandular epithelial compartment has undergone EMT to support the growth of the lesion.

Endometriosis is an estrogen‐dependent disease. Since estrogen induces EMT in other diseases including ovarian cancer and breast cancer^77^, ^78^, ^79^ it is likely to drive EMT in endometriosis. Estrogen increases ZEB1 promoter activity and mRNA expression in Ishikawa cells in vitro,^80^ which had downstream effects on mRNA expression of E‐cadherin (decreased) and vimentin (increased). In another study using primary endometrial epithelial cells, treatment with β‐estradiol led to a decrease in E cadherin protein expression and an increase in migratory and invasive properties.^69^ In the same study, treatment with an estrogen receptor antagonist ICI increased E cadherin expression and decreased vimentin and Snail mRNA expression,^69^ reversing EMT.

While estrogen may drive EMT in endometriosis, progesterone resistance also plays a part in disease pathogenesis. A recent study has shown that EMT may contribute to the downregulation of progesterone receptors (PR) in lesions, making them less responsive to progestin therapy.^81^ In this study, knockdown of SNAI1 and SNAI2 in endometriosis cell lines resulted in an increase in PR expression^81^ indicating that PR resistance may be driven via EMT.

### Immune cells

Immune cells, from both the innate and adaptive immune responses, play a role in endometriosis (Figure 1), both those shed in eutopic endometrium and those in the peritoneal environment are thought to contribute to disease pathogenesis.

#### The innate immune response in endometriosis

scRNAseq of eutopic endometrium revealed 13 transcriptomically distinct immune cell subtypes,^82^ 5 from a macrophage cluster and 8 from a lymphocyte cluster. Monocyte and macrophage enrichment scores were elevated in mid secretory endometrium in endometriosis patients compared to controls.^82^ This supports an earlier study where immunohistochemistry of eutopic endometrium showed that CD68^+^ macrophages were increased in the endometrium of endometriosis compared to control patients.^83^ This coincides with an increase in eutopic endometrial monocyte chemoattractant protein‐1 (MCP‐1) expression in endometriosis patients.^84^ The importance of eutopic endometrial‐derived macrophages to lesion establishment and progression was demonstrated in small animal models.^85^, ^86^ Depletion of eutopic endometrial macrophages by doxycycline in a donor‐recipient mouse model resulted in fewer lesion‐derived macrophages and smaller lesions.^86^

Mass cytometry of peritoneal fluid from endometriosis patients reveals 40 distinct immune cell types^87^ which were stratified by disease stage. Macrophages exhibited an increase in both pro‐inflammatory (CD64/CD40) and anti‐inflammatory (CD163/CD206) markers compared to patient matched peripheral blood, indicating alternative activation of macrophages. This supported an earlier study where scRNAseq of peritoneal fluid from endometriosis patients identified seven subsets of macrophages,^88^ in which both pro‐inflammatory and pro‐repair genes were expressed, highlighting the heterogeneity of macrophages and their potential dual roles in the pathogenesis of endometriosis.

#### Adaptive immune response in endometriosis

Mass cytometry also indicated that T cell activation was increased in the peritoneal fluid of patients with endometriosis patients compared with controls.^87^ CD25^high^Foxp3^+^ regulatory T cells (Tregs) were increased in peritoneal fluid in endometriosis,^89^ and positively correlated with increases in peritoneal cytokines, IL‐10 and TGFB1. Both cytokines regulate Fibrinogen‐like protein 2 (FGL2) expression, and peritoneal fluid Tregs of endometriosis patients also have increased FGL2 expression.^90^ FGL2 drives macrophage polarization toward the pro‐repair phenotype highlighting a potential positive feedback loop between T cells and macrophages that drives endometriosis progression. NK cell cytoxicity is decreased in the peritoneal fluid of endometriosis patients^91^ irrespective of disease stage, indicating a mechanism by which menstrual fragments may survive in the peritoneal cavity.

### Altered genomic programs

#### Genetic risk factors

Twin studies have estimated the heritability of endometriosis at 0.47–0.51^92^, ^93^ indicating that genetic factors contribute to 50% of the variation in disease risk. Genome‐wide association studies (GWASs) investigating the association between common germline genetic variants and endometriosis have provided strong evidence for the contribution of many genetic variants across the genome^30^, ^94^, ^95^, ^96^, ^97^ (Figure 1). The most recent published endometriosis GWA meta‐analysis^97^ identified 19 independent signals in 14 genomic loci associated with the disease.^97^ Candidate genes in the risk regions have been linked to hormonal regulation (*ESR1*, *FSHB*, *GREB1*) and cell adhesion and proliferation (*CDC42*, *CDKN2BAS*, *VEZT*, *FGD6*). The estimated proportion of variance in endometriosis risk captured by common single nucleotide polymorphisms (SNPs) across the genome (SNP‐based heritability) was 26%.^96^, ^98^ When restricted to more severe forms of the disease (rAFS Stage III/IV) the variance captured increases to 34%.^96^ Similarly, studies report larger genetic effects and a larger genetic burden for individual risk factors in more severe disease,^99^ with lead GWAS SNPs estimated to capture 5.19%^97^ of the variance in stage III/IV disease compared to 1.75% in overall disease risk.^97^ Identification of candidate causal genes in endometriosis risk loci relies on subsequent functional annotation of variants in these regions.

#### Somatic mutations

Evidence is emerging that endometriotic lesions display a mutational burden higher than expected in normal tissue through the acquisition of mutations as cells age.^28^ Untargeted sequencing approaches have identified mutations across the genome, some being cancer driver genes (Table 1),^26^, ^28^, ^108^ suggesting they may provide an advantage in cell attachment, growth or survival thereby contributing to endometriosis pathogenesis (Figure 1). A profile of mutated cancer driver genes in endometriotic lesions is beginning to emerge (Table 1).

**TABLE 1:** Somatic mutations in cancer driver genes reported in endometriotic lesions and human endometrium.

Gene	SUP	OMA	DIE	Endometrium	References
*AKT1*				Yes	169
*ARHGAP35*		Yes		Yes	28, 110
*ARID1A*	Yes	Yes	Yes	No	26, 108, 169, 170, 171
*ARID5B*				Yes	110
*ATM*				Yes	110
*ATRX*			Yes	Yes	111
*BRAF*		No		Yes	110, 171
*CARD10*		Yes			172
*CARD11*		Yes			172
*CDH4*				Yes	110
*CDKN1B*				Yes	110
*CREBBP*				Yes	110
*CSMD3*	Yes				28
*CTCF*		Yes			173
*CTNNB1*				Yes	108
*DNAH7*			Yes		111
*EGFR*				Yes	110
*ERBB2*	Yes		Yes	Yes	108, 110
*ERBB3*			Yes		110
*ERK1*		No			171
*ERK2*		No			171
*ERRB2*				Yes	169
*FAT1*				Yes	110
*FBN2*		Yes			28
*FBXW7*		Yes		Yes	28, 110
*FGFR2*				Yes	110, 169
*FOXA2*				Yes	110
*FRG1*		Yes			28
*HEATR1*		Yes		No	28
*HRAS*		No		Yes	110, 171
*KIAA1109*		Yes			28
*KMT2C*				Yes	110
*KMT2D*				Yes	110
*KRAS*	Yes	Yes	Yes	Yes	26, 28, 108, 110, 111, 169, 171, 174, 175
*MUC6*		Yes			28
*NF1*				Yes	110
*NOTCH2*				Yes	110
*NRAS*		No		Yes	169, 171
*PIK3CA*	Yes	Yes	Yes	Yes	26, 28, 108, 110, 169, 171
*PIK3R1*		Yes		Yes	28, 110
*PLCG1*				Yes	110
*PLXNB2*		Yes		Yes	28
*PPP2R1A*		Yes	Yes	Yes	26, 28, 110, 171
*PRDM1*				Yes	110
*PTEN*		No	Yes	Yes	108, 110, 169, 171
*PTPN13*		Yes			28
*RRAS*				Yes	110
*RYR1*			Yes	Yes	111
*SMAD2*				Yes	110
*SPOP*				Yes	110
*STAG2*				Yes	110
*TASR31*		Yes			28
*TP53*				Yes	110
*TRERF1*		Yes			176
*TTN*		Yes			28
*ZFHX3*				Yes	110

*Note*: Yes indicates mutation has been detected in at least one patient in the referenced study. No indicates no mutation has been identified and specifically reported in the referenced study. Blanks represent not specifically mentioned.

Abbreviations: DIE, deeply infiltrating endometriosis; OMA, endometrioma; SUP, superficial peritoneal endometriosis.

The suite of mutations appears relatively consistent across lesion subtypes, despite slight variations. The genes most commonly mutated in endometrioma (OMA) include KRAS, PIK3CA and TTN^28^ whereas KRAS and PTEN are more common in deep infiltrating endometriosis (DIE)^26^, ^108^ (Table 1). Current evidence suggests a similar profile will exist in superficial (SUP) lesions.^108^ Significantly, separation of epithelial and stromal compartments revealed that mutations are restricted to the epithelial glands,^109^ suggesting that the influence of acquired genetic mutations on endometriosis pathogenesis is mediated by the epithelial cells directly or through their interaction with surrounding cells and microenvironment.

Mutational profiles of epithelial glands display heterogeneity. Examination of individual epithelial glands both within endometriotic lesions, and individual glands from different lesions in the same patient identified variations in their mutational profile.^28^ In endometriotic glands of one subject, similar PIK3CA mutations were observed across six glands, whereas different and distinct mutations were found in the ovaries of another patient,^28^ indicating different lesions harbor distinct mutation profiles and may be populated by a unique set of cells. Clonality analysis suggested a selective advantage existed in some glands.^28^ The contribution of mutations to endometriosis pathogenesis may be regulated by the mutation and from when and where it was acquired.

Recently epithelial cells of the normal endometrium were shown to harbor an elevated mutational burden, ranging from 209 to 2833 base substitutions per woman,^110^ many in cancer driver genes (Table 1). In an individual, the mutational profile of the endometrium is similar although not identical to endometriotic lesions, suggesting glands are the source of the initial mutations and that lesions acquire additional mutations, particularly in cancer driver genes when matched DIE lesions were compared to eutopic endometrium.^111^

Only a few of many endometrial glands can be examined experimentally, and they also show significant heterogeneity in mutational profiles within the same patient.^110^ Three‐dimensional profiling and tissue clearing techniques (Figure 1) found that vertical functionalis glands with matching mutational profiles occupy endometrial regions up to 4.7 mm^2^, which originated from a common section of a horizontal rhizome‐like gland in the basalis.^39^ Thus, endometrial glands in distal regions are populated by progeny of common epithelial progenitor cells. Endometrial stem/progenitor cells in the rhizome‐like structures of basalis endometrium that acquire somatic mutations in cancer driver genes may confer a selective advantage for their survival, attachment and ability to establish lesions if they reach the peritoneal cavity via retrograde menstruation, thereby contributing to endometriosis pathogenesis. A number of questions, remain: how and when these mutations are acquired, how are they influenced by the surrounding environment, do they achieve a selective advantage and achieve clonality and how do they avoid a progression to malignancy?

#### Environmental contribution to endometriosis and potential links to mutation

Somatic single‐nucleotide variants, cytogenetic aneuploidy and structural chromosomal variants are strongly correlated with age,^110^, ^112^ suggesting both time and environment contributions to accumulation of DNA variations (Figure 1). The rate of mutation acquisition differs among cells, tissue and individuals and is primarily associated with relative exposures to environmental factors, inherited deficits in DNA‐repair systems and acquired genetic and epigenetic abnormalities.^113^ Environmental damage can be organ and cell‐specific and induced by lifestyle factors including ultraviolet light, ionizing radiation, tobacco smoke, chemotherapeutic drugs, and exposure to environmental toxins.^113^

Environmental and lifestyle factors have been investigated in endometriosis etiology. Smoking,^114^ alcohol consumption^115^ and dietary choices do not show robust associations with endometriosis, although some evidence suggests excess red meat intake^116^, ^117^ and a protective effect of phytoestrogen intake.^118^, ^119^ Nor does there appear robust evidence for an association between environmental pollutants and endometriosis. Dioxins and polychlorinated biphenyls showed no association,^120^, ^121^, ^122^, ^123^ with limited and contradictory evidence for bisphenol A,^124^, ^125^ phthalates^125^, ^126^ and parabens.^127^, ^128^

Despite current estimates suggesting a 49% environmental contribution to endometriosis risk, there is currently limited data supporting specific environmental exposures or lifestyle factors increasing the risk of endometriosis, and less suggesting they influence the induction of mutations in endometrial cells. Most studies are limited by sample size and variations in environmental conditions experienced by the different populations and are yet to directly assess whether environmental exposures increase the mutational burden in relevant cell types. The emerging evidence for elevated cancer driver mutations present in benign endometriosis, and their clear association with age and experience suggest this could represent a rich source of enquiry for endometriosis pathogenesis. The increasing availability of deeply phenotyped patient cohorts (Figure 1) and complex 3D in vitro models (Figure 1) now provides opportunities to explore environmental and lifestyle contributions to endometriosis through a direct influence on the DNA of endometrial cells.

#### Genetic regulation of gene expression

Studies investigating changes in gene expression associated with endometriosis have identified large differences in gene expression between eutopic endometrium and ectopic endometriosis lesions.^129^, ^130^, ^131^ Deregulated genes in endometriotic lesions were enriched in PI3K‐AKT, WNT and MAPK signaling, oxidative stress and focal adhesion pathways and included several genes from the IGF/IGFBP and MMP families. Whether or not the dysregulation of these pathways occurs as a cause or consequence of disease remains to be determined. Differences in the expression of candidate endometriosis susceptibility genes have also been identified between eutopic endometrium from patients with and without endometriosis^132^, ^133^ however, these genes do not replicate in larger genome‐wide studies.^134^, ^135^, ^136^

Statistical approaches have identified putative causal relationships between genetic variants, expression of genes and risk of endometriosis, through the integration of summary statistics from GWAS and expression quantitative trait loci (eQTLs)^137^ (Figure 1). Such approaches identified that variants regulating the expression of genes involved in cell adhesion and proliferation, *LINC00339*, *VEZT*, *FGD6*, and *CDC42*, in endometrium and blood, also increase risk of endometriosis.^134^, ^135^, ^138^ Functional annotation of variants on chr12q22, regulating expression of *VEZT* and *FGD6*, revealed that the causal variant likely resides in a bidirectional promoter for these two genes and has also been associated with risk of epithelial ovarian cancer.^139^ Functional studies investigating the interaction between genetic variants on chr1p36.12 and nearby genes suggested that endometriosis risk variants interact with the promoters of *LINC00339*, *CDC42* and *WNT4* and the risk allele is associated with increased expression of CDC42 in blood cells.^138^, ^140^ Expression of genes with critical roles in hormonal regulation have also been implicated in endometriosis. Variants on chromosome 6 near *ESR1* have been associated with endometriosis and various other reproductive traits and diseases. While no evidence of risk variants regulating expression of genes in this region has been reported, genes in the region are highly correlated with the expression of *ESR1* and *PGR* suggesting genetic variants in the region could impact co‐regulation of these hormone receptor genes.^141^ Risk variants located in another estrogen responsive gene, *GREB1*, have been associated with transcriptional splicing of *GREB1* in ovarian tissue.^142^ Large‐scale eQTL and sQTL studies^142^, ^143^ provide strong evidence for tissue and cell‐type specific genetic effects on gene expression and splicing, highlighting the need to investigate genetic effects in disease relevant tissues and cell‐types to better understand how genetic risk factors regulate genes and increase endometriosis risk.

#### Epigenetic modifications

Epigenetic modification refers to changes in gene activity that do not arise from changes in DNA sequence, but rather are due to behaviors and environmental exposures (Figure 1). DNA methylation (DNAm) is one of the most common modifications measured in disease studies. Methylation studies in human endometrium and endometriosis have identified differences at DNAm sites across the genome between endometriotic and normal endometrial tissue^136^ and stromal cells.^144^ Differentially methylated sites were mapped to genes and pathways implicated in the pathology of endometriosis and decidualization including HOX gene clusters, nuclear receptor genes, the GATA family of transcription factors,^144^ *WNT* signaling, angiogenesis, cadherin signaling, and gonadotropin‐releasing‐hormone‐receptor pathways.^136^ Hypomethylation and overexpression of GATA6 in ectopic endometrial stromal cells restricts the ability of cells to decidualise and has been linked to the transformation of endometrial stromal cells into endometriotic‐like cells that produce estrogen.^145^ Changes in methylation profiles across the menstrual cycle in eutopic endometrium from patients with endometriosis have also been reported however, these changes fail to replicate between studies likely due to small sample sizes and limited power to detect subtle differences.^136^, ^146^, ^147^ Epigenetic signals capture variation in disease however, it can be challenging to distinguish between cause and consequence of disease. Epigenetic profiles vary widely between tissues and cell‐types,^148^ as such cell‐type specific epigenetic effects associated with endometriosis may be relevant to disease etiology and pathogenesis.

Evidence for putative causal effects of methylation on endometriosis can be discerned from genetics. Genetic effects on methylation in human endometrium have been identified in the form of methylation quantitative trait loci (mQTLs). Variants regulating methylation in endometrium have been associated with reproductive traits and diseases including a variant regulating methylation in *GREB1* and endometriosis.^147^

#### Bacterial contamination hypothesis

The association between endometriosis and chronic inflammation may be explained by the “bacterial contamination hypothesis” where bacterial endotoxin, that is, lipopolysaccharide or LPS induce the pro‐inflammatory environment via the LPS/TLR4 cascade in the pelvis.^149^ Cultured menstrual blood is more highly contaminated with *Escherichia coli* from women with endometriosis than those without. Furthermore, higher levels of endotoxin are found in menstrual and peritoneal fluid in women with endometriosis.^150^ The increased endotoxin in the pelvic area of women with endometriosis may result from migration of *E. coli* from the vagina to the uterine cavity via the menstrual blood, or from the *E. coli* and endotoxin arising from the gut and translocating via enterocytes into the pelvic cavity.^150^ The growth of endometriotic lesions is stimulated by TLR4‐mediated inflammation induced by *E. coli*.^150^ Higher levels of prostaglandin E2 in menstrual fluid of women with endometriosis also enhances the growth of *E. coli* in vitro^151^ and increased microbial colonization and endometritis is seen in the uterus of women with endometriosis.^152^ Analysis of bacterial ribosomal RNA genes revealed an increased bacterial subclinical infection risk in women with endometriosis compared to those without.^153^ Potential treatment strategies for controlling bacterial colonization include antibiotic treatment where a single dose of the antibiotic Levofloxacin decreases some bacterial genera in the endometrium of women with and without endometriosis.^100^ Whether or not these bacterial genera are contributing to the cause or more likely the progression of endometriosis is yet to be determined. While there is evidence of a dysregulated gut or reproductive tract microbiome in women with endometriosis, there is little consensus regarding the particular microbiota that may lead to endometriotic lesion growth.^101^ Additionally, some bacteria promote endometriosis and others potentially protect against it highlighting the need for further investigations into this area.^102^

## INFLUENCE OF NEW TECHNOLOGIES ON UNDERSTANDING ENDOMETRIOSIS PATHOGENESIS

### Organoids and multicellular organoids

The development of 3D organoid cell culture models using patient‐derived cells allows the investigation of in vivo mechanisms in a pre‐clinical setting (Figure 1). Endometrial epithelial organoids (EEO) have been established from human and mouse endometrial epithelia and comprise ciliated and unciliated, proliferating, secretory and stem/progenitor epithelial cells.^103^, ^104^, ^105^, ^106^ EEO reflect the cycling human endometrium by responding to hormones of the menstrual cycle including estrogen and progesterone, as evident in their gene and protein expression, and cellular changes.^103^, ^104^, ^105^, ^106^ EEO retain a “memory” of the donor as shown in organoids derived from endometriosis and endometrial cancer patients.^107^ Indeed, EEO from ectopic and eutopic endometriosis samples express epithelial cell markers and steroid hormone receptors, preserving endometrial glandular structure and cell characteristics. Endometriosis‐derived organoids enable comprehensive investigations into the development and pathogenesis of endometriosis. Findings from stage IV endometriosis‐derived organoids suggest involvement of cancer driver genes in the development of endometriosis.^107^ Lesion formation can be modeled by implanting ectopic endometriosis‐derived organoids in the peritoneal cavity of mice.^107^ The role of epigenetics in the development of endometriosis has been investigated using organoids derived from ectopic and eutopic endometrium from endometriosis patients.^154^ Recently, menstrual fluid organoids have been established which reflect the same properties as those derived from endometrial tissue.^155^, ^156^ The efficiency and non‐invasive method for obtaining endometrial cells from menstrual fluid presents a promising avenue for personalized medicine and tailoring drug treatments for individual endometriosis patients.

While endometrial epithelial organoids have many benefits in recreating a 3D in vivo environment, endometriosis is a multicellular disease involving interactions between epithelial cells, stromal fibroblasts, extracellular matrix, immune cells, vasculature and nerve cells. Endometrial stromal cells from women with endometriosis exhibit disordered decidualization, a process essential for establishing a receptive endometrial environment for embryo implantation.^157^, ^158^, ^159^ As such, incorporating these cells into a 3D model of endometrial epithelial organoids would provide greater insight into the effect of endometriosis on both the eutopic and ectopic endometrium. Recent advances have been made in establishing an extracellular matrix and suitable media conducive to both stromal and epithelial growth in co‐culture.^160^, ^161^, ^162^ Multicellular 3D culture systems are needed to appropriately model in vivo endometriosis conditions in vitro.

### Single cell RNA sequencing

The initial application of single‐cell RNA‐seq to endometriosis focused on generating cell atlases of spatiotemporal time points of the endometrium (Figure 1). One study assessing individual samples from successive days of the menstrual cycle identified four major transformations of the endometrium during the menstrual cycle and the interplay of cell types mediating these transitions.^163^ Subsequently, analysis has focused on the maternal‐fetal interface and the cell types present. This resulted in the identification of two perivascular cells, distinguished by different *MCAM* expression concentrations^164^ and three stromal cell subsets, two of which expressed markers similar to decidualized stromal cells identified earlier, and three NK cells subsets suggesting an immune modulatory component at this interface. A similar analysis reported two distinct endometrial fibroblasts, smooth muscle and endothelial cells, epithelial and two distinct NK cells with 48.7% of the decidual sample containing cells with high ECM expression and decidualized fibroblasts displaying two distinct differentiation trajectories.^165^, ^166^

The identification of these cells and the measurement of their individual gene expression profiles in multicellular organs provides the opportunity to chart which cells are present and determine whether their relative proportions change in individual patients or correlate with clinical presentations. Additionally, the characterization of their distinct transcriptomic profiles provides the opportunity to identify whether subtle variations within cell types occur that are indicative of pathogenic processes. Together the data generated by in‐depth single‐cell sequencing provides the powerful and unique opportunity to chart all disease‐relevant cells within the analyzed tissue and their potential individual contributions to endometriosis etiology.

While this powerful technique is generating an atlas of the endometrium in its varying spatio‐temporal presentations, it is yet to be comprehensively utilized in the discovery of variations related to endometriosis. Leveraging single‐cell RNA‐seq data from the endometrium of patients without endometriosis, bulk RNA‐seq data from patients with and without endometriosis, and cellular deconvolution showed an increased enrichment of epithelial and endothelial cells, plasmacytoid dendritic cells and monocytes in the endometrium of patients with endometriosis during the mid‐secretory stage.^82^ However, this study was limited by sample size and the accuracy of cell deconvolution methods from bulk RNA‐seq data. Recently, a study using 19 individuals identified a mesenchymal cell signature derived through altered differentiation that was more likely present in women with endometriosis.^167^ These cells showed changes in growth profiles, were characterized by high expression of matrix metalloproteinases (MMP) *MMP3* and *MMP10*, and may have a role in endometriosis etiology. A similar fibroblast signature characterized by *MMP3* and *MMP10* was observed in menstrual fluid of patients with endometriosis,^168^ suggesting these altered fibroblasts may be maintained during menstruation and may represent a possible non‐invasive diagnosis signature.

## IMPLICATIONS FOR DIAGNOSIS AND NEW THERAPIES

The bringing together of recent major advances in technologies available to investigate endometriosis; next generation sequencing, new analytical methods, endometrial stem/progenitor cell identities and organoids has major implications for finding a non‐invasive diagnostic test desperately needed for endometriosis. Menstrual fluid provides non‐invasive sampling of endometrial tissue for somatic mutations, endometriosis risk genes, endometrial stem/progenitor cells and endometrial proteins as potential biomarkers for diagnosis. Menstrual fluid‐derived and endometrial tissue organoids provide a platform for modeling molecular mechanisms involved in endometriosis. As the pathogenesis of endometriosis is revealed in future studies, these organoids can be used for drug screening and provision of personalized medicine to patients with endometriosis. New concepts on the etiology of endometriosis are emerging, including the “cells of origin” and their transport into the pelvic cavity, genetic risk factors, epigenetic modifications and somatic mutations, environmental factors such as bacterial contamination and EMT. Combining this knowledge with new high powered molecular and cellular technologies will likely provide new avenues for diagnosis and treatment of endometriosis based on the etiology of endometriosis as it becomes revealed.

## CONFLICT OF INTEREST

The authors declare no conflict of interests for this article.

## Data Availability

Data sharing not applicable to this article as no datasets were generated or analysed during the current study.
